# Reproductive Health-Related Knowledge, Attitude, and Practices in Women of Reproductive Age in Underdeveloped Areas of Punjab, Pakistan

**DOI:** 10.7759/cureus.31043

**Published:** 2022-11-03

**Authors:** Murtaza Sharif, Hafiza Kiran Majeed, Kanwal Tagar, Sonam Lohana, Asma Rauf, Mujtaba Sharif, Khadija Jadun, Widhya Devi, Abeel Naseer

**Affiliations:** 1 Community Medicine, Services Institute of Medical Sciences, Lahore, PAK; 2 Obstetrics and Gynaecology, Faisalabad Medical University, Faisalabad, PAK; 3 Internal Medicine, Chandka Medical College, Larkana, PAK; 4 Internal Medicine, Liaquat University of Medical and Health Sciences, Jamshoro, PAK; 5 Cardiology, Bilal Hospital, Rawalpindi, PAK; 6 Obstetrics and Gynaecology, Qiqihar Medical University, Qiqihar, CHN; 7 Pediatric Medicine, Women Medical and Dental College, Abbottabad, PAK; 8 Internal Medicine, Isra University, Hyderabad, PAK; 9 Internal Medicine, Nawaz Sharif Medical College, Gujrat, PAK

**Keywords:** contraception, menstrual irregularities, child bearing age, dijikot, reproductive health problems, reproductive health

## Abstract

Background: In addition to physical welfare, reproductive health is also vital for psychological well-being. All stages of reproduction can take place safely if reproductive health is well cared for, and it ultimately leads to the formation of healthy new offspring. The aim of this study is to know about reproductive health-related knowledge and practices in women of reproductive age in an underdeveloped area in Pakistan and to identify the associated factors that give a meaningful impact on reproductive health.

Methods: A cross-sectional study was carried out among women of childbearing age in underdeveloped areas in the province of Punjab, Pakistan. A sample of 400 was taken on a random basis. All the relevant data were collected from February 1, 2022, to August 30, 2022, with the help of a structured questionnaire, designed specifically for the study, informed consent was taken from all of the participants before data collection. Questions were asked about their menstrual cycles, use of contraceptives, knowledge about sexually transmitted diseases, screening of cervical cancer, pap test, human papillomavirus vaccine, and related to home or hospital deliveries. Socioeconomic classes were defined by different income ranges per month as lower class, upper lower class, middle class, upper middle class, and upper class.

Results: Ten percent of participants with education up to fifth grade have never used any method of contraception while 70% of participants who studied up to eighth grade never used the same. In lower class and upper lower class, the prevalence is 33.3% and 41.7%, respectively. The prevalence of screening for cervical cancer is 50% in married women and 60% in the upper middle class. Of women with education up to eighth grade, 65% answered with No, and the prevalence is 50% for lower-class women. Regarding the human papillomavirus vaccine, 41.7% of married women, 33.3% of women in upper class, and 50% of women in the middle class mentioned that they know about it, while 68.4% of women have education up to eighth grade and 47.4% of lower-class women answered with No. Of women with education up to eighth grade, 92.5% had one to two deliveries at home, and 68.8% of women with education up to fifth grade had three to four deliveries at home. Fifty percent of women from both lower and upper lower classes had one to two deliveries at home. Twelve women from the upper middle class had all of their deliveries at home and 20 had five to six deliveries at home. Of women with education up to fifth grade and eighth grade, 64.3% and 28.6%, respectively, had their all deliveries at a hospital; 22.9% of women from the upper class and 20% of the upper middle class also had all deliveries at the hospital, Thirty-three women who graduated from college had one to two deliveries in the hospital. All of these results are found to be significant with a p-value <0.05.

Conclusion: Knowledge about reproductive health is less prevalent in women with low education and the same is for lower and lower middle socioeconomic class. The education level of women and their socioeconomic class is one of the major factors that have a meaningful impact on their reproductive health and practices.

## Introduction

More than 20% of the burden of disease among women of reproductive age is connected with sex and reproduction. In the developing world, a woman’s lifetime risk of death from maternal causes is 33 times that of her counterparts in developed countries [1]. It is also recognized that women suffer silently from a large number of reproductive illnesses, which were termed the silent emergency. This understanding leads to women’s health researchers and activists focusing more on women’s health, especially in the field of reproductive health [2]. Women of reproductive age have health problems like endometriosis, uterine fibroids, gynecologic cancer, HIV AIDS, interstitial cystitis, polycystic ovary syndrome (PCOS), menstrual irregularities, complications related to contraceptive use, mid-cycle pain, mid-cycle spotting, dysmenorrhea [3]. 

The major problem of reproductive health that is faced by three fourth of the world's women, i.e., menstrual irregularities, result from various disorders including endometriosis, fibroids, PCOS, hormonal imbalance, increased stress levels, and improper use of contraceptives [4]. A study showed that malnutrition in females, e.g., due to anorexia nervosa and bulimia nervosa, which leads to reduced energy levels affects up to 5% of women of reproductive age causing amenorrhea, infertility, and increased likelihood of miscarriage [5].

An estimated 105 million married women in the developing world face an unmet need for contraception [6]. The fear of weight gain affects the uptake and continuation of hormonal contraceptives, although existing trials indicate that any such effects are small. For all methods of hormonal contraception, weight above 70 kg is associated with increased failure rates [7]. Evidence suggests that excess insulin levels lead to high androgens level, which suppresses ovulation and causes excessive hair growth and acne, two important signs of PCOS [8].

Evidence indicates that depression is closely linked with disproportionate exposure to risk factors, stressful life events, and adverse life experiences that are more common for women and that also affect their reproductive health [9]. Mental disorders including depression, which is the most important mental health condition for women worldwide, make a significant contribution to the global burden of disease. Women suffer more often than men from the common disorders of depression and anxiety, both on their own and as comorbidities [10]. According to a study, 61.6% of women globally are suffering from major mental disorders including depression and anxiety, leading to increased reproductive health problems [11].

An estimate suggests that 25% of women of reproductive age have menstrual irregularities including heavy bleeding in Turkey; this is 20% in China and 15% in India. Infertility, defined as not getting pregnant within 12 months of having unprotected sex with the same partner, affected 5.8% of married or cohabiting women aged 15-44 years in 2008-2012 in the United States [12]. Worldwide, in 2015, 12% of women are estimated to have had an unmet need for family planning; that is, they wanted to stop or delay childbearing but were not using any method of contraception [13]. 

## Materials and methods

Study design, period, and area

This is a community-based cross-sectional study conducted for a period of seven months from February 1, 2022, to August 30, 2022, in three villages around Dijikot in the Punjab province of Pakistan.

Inclusion and exclusion criteria

Mentally and physically healthy women between the ages of 18-48 years. Women who were under or above the aforementioned age range or/and had congenital deformities, intellectual disabilities, a history of major accidents or trauma, and a family history of motor neuron disease were excluded from this study.

Study population

The total population of villages covered by the study is estimated to be 105,052, of which 54,627 are males and 50,425 are female. From this female population, those who are less than 18 years old and more than 48 years old (N=12,316) were excluded, and 38,109 females under the reproductive age group were listed.

Sample size and sampling technique

A multistage random sampling technique was used to select villages, and each village is considered a cluster. The population proportion to size method was used in selecting the samples from each cluster. With a 95% confidence interval, 4.88% margin of error, a population size of 38,109, and 50% response distribution for each question, the sample size calculated using the formula n =N x/((N-1)E2 + x) was 400.

Study tool

A structured questionnaire consisting of three sections was used. The first section (with six questions) covered the demographic data of the woman, including her current age, educational status, socioeconomic status based on family joint income, type of family, marital status, and religion. The second part (with 10 questions) consisted of the age of menarche start, duration of the menstrual cycle, number of days of menstrual bleeding, number of painful bleeding days, irregular bleeding episodes in a menstrual cycle, age of first sexual encounter, number of pregnancies and parity, number of sexual partners, and age of marriage. The third section had 11 questions related to awareness regarding reproductive health and hygiene practices. Socioeconomic classes are defined by different income ranges per month. Monthly income levels of Rs 4,000 (Pakistani rupee) to Rs 20,000 was defined as the cut-off for the lower class, and Rs 20,000 to Rs 40,000 for the upper lower class. For the middle class, the cutoff was Rs 40,000 to Rs 80,000 and for the upper middle class, the cutoff was Rs 80,000 to Rs 100,000. People earning above Rs 100,000 were categorized as belonging to the upper class.

Statistical analysis

The data collected were entered in Microsoft Excel (Microsoft Corporation, Redmond, Washington, United States), and statistical analysis was done using IBM SPSS Statistics for Windows, Version 28.0 (Released 2021; IBM Corp., Armonk, New York, United States). The Chi-square test was applied, and p-value < 0.05 was considered significant. 

Ethics statement

The study protocol was approved by the Ethics Committee of Faisalabad Medical University, Faisalabad, Pakistan (approval number 2022/8-140). All patients were informed about the objectives of the studies. They provided their consent and confidentiality was assured among participants regarding the information they give for this research article. The study was conducted in line with the ethical principles of the Declaration of Helsinki.

## Results

In this study, the majority of the respondents were between 22-28 years of age (33%), with education mostly up to eighth grade (52%), unmarried (85%), from the lower class (40%), having a nuclear type of family (60%), and belonged to the Muslim religion (80%) (Table 1).

**Table 1:** Distribution of participants according to selected sociodemographic characteristics

Category		Number	Percentage
Age of respondent (years)	15-21	83	20.8%
	22-28	132	33.0%
	29-35	67	16.8%
	36-42	67	16.8%
	43-48	51	12.8%
Education of respondent	up to 5th Grade	116	29.0%
	Up to 8th Grade	208	52.0%
	Matriculation	0	0.0%
	Intermediate	43	10.8%
	Bachelor's degree (college graduation)	33	8.3%
Marital status	Married	40	10.0%
	Unmarried	340	85.0%
	Divorced	20	5.0%
Socioeconomic class	Upper	32	8.0%
	Upper Middle	48	12.0%
	Lower Middle	60	15.0%
	Upper Lower	100	25.0%
	Lower	160	40.0%
Religion	Muslim	320	80.0%
	Christian	20	5.0%
	Hindu	48	12.0%
	Others	12	3.0%
Type of family	Nuclear	240	60.0%
	Joint	100	25.0%
	Three Generation	60	15.0%

Table 2 describes the menstrual evaluation; 60% had menarche at the age of 12 years, 77% had a menstrual cycle of 25 to 28 days, 70.5% had four to five days of menstrual bleeding, and 14% had at least one episode of irregular bleeding in a menstrual cycle.

**Table 2:** Prevalence of menstrual status in study participants

		Frequency	Percentage
Age at menarche	11 years	80	20.0%
	12 years	240	60.0%
	13 years	48	12.0%
	14 years	20	5.0%
	15 years	12	3.0%
Duration of menstrual cycle	do not know	16	4.0%
	21-24 days	4	1.0%
	25-28 days	308	77.0%
	29-32 days	64	16.0%
	33-36 days	8	2.0%
Days of menstrual bleeding	do not know	20	5.0%
	4 to 5	282	70.5%
	6 to 7	70	17.5%
	8 to 9	20	5.0%
	10 to 11	8	2.0%
Days of painfull menstrual bleeding	do not know	36	9.0%
	2 to 3	240	60.0%
	4 to 5	112	28.0%
	7 to 8	8	2.0%
	9 to 10	4	1.0%
Irregular bleeding episodes in a mentrual cycle	0	280	70.0%
	1	56	14.0%
	2	40	10.0%
	3	16	4.0%
	4	8	2.0%

Regarding the age of the first sexual encounter, 60% of participants did not answer on ethical grounds while 22% had their first sexual encounter at the age of 12 to 16 years, and 3.75% (n=15) got married at 15 to 22 years of age. Forty percent had three to four pregnancies and 50% had two to three live births (excluding miscarriages) at the time of this study.

**Table 3:** Demographies related to sexual practice of participants

		Frequency	Percent
Age of first sexual encounter (years)	Can not tell	240	60.0%
	12-16	88	22.0%
	17-21	40	10.0%
	22-26	20	5.0%
	27 or onward	12	3.0%
Age of marriage (years)	Never married	340	85%
	15-22	15	3.75%
	23-30	20	5%
	31-37	18	4.5%
	38-46	7	1.75%
Number of pregnancies	1-2	28	7.0%
	3-4	160	40.0%
	5-6	120	30.0%
	7-8	80	20.0%
	9-10	12	3.0%
Parity	0-1	8	2.0%
	2-3	200	50.0%
	4-5	120	30.0%
	6-7	40	10.0%
	8-9	32	8.0%
Number of sexual partners	do not know	240	60.0%
	1	140	35.0%
	2	12	3.0%
	3	8	2.0%
	4	0	0.0%

Thirty-three percent of women aged 22-28 years never used any method of contraception and combined oral contraceptive pills usage was found to be 31.7% in the same age group (Table 4). It was found that 10.8% of participants with education up to fifth grade and 70% of participants with education up to eighth grade have never used any method of contraception. In lower class and upper lower class, 33.3% and 41.7%, respectively, never used any form of contraceptive. These results are found to be significant with a p-value < 0.05 (Table 5).

**Table 4:** Prevalence of different methods of contraception used by participants COCP: combined oral contraceptive pills; IUDs: intrauterine device system

Population characteristics		Method of contraception used									
		Never used		COCPs		IUDs		Depot injections		Barrier (condoms, diaphragm)	
		Frequency	Percentage	Frequency	Percentage	Frequency	Percentage	Frequency	Percentage	Frequency	Percentage
Age of respondent	15-21	56	23.3%	10	16.7%	7	17.5%	1	20.0%	9	16.4%
	22-28	80	33.3%	19	31.7%	14	35.0%	1	20.0%	18	32.7%
	29-35	40	16.7%	10	16.7%	6	15.0%	1	20.0%	10	18.2%
	36-42	40	16.7%	11	18.3%	6	15.0%	1	20.0%	9	16.4%
	43-48	24	10.0%	10	16.7%	7	17.5%	1	20.0%	9	16.4%
Education of respondent	up to 5th Grade	26	10.8%	20	33.3%	40	100.0%	0	0.0%	30	54.5%
	Up to 8th Grade	168	70.0%	40	66.7%	0	0.0%	0	0.0%	0	0.0%
	Matriculation	0	0.0%	0	0.0%	0	0.0%	0	0.0%	0	0.0%
	Intermediate	13	5.4%	0	0.0%	0	0.0%	5	100.0%	25	45.5%
	Bachelor	33	13.8%	0	0.0%	0	0.0%	0	0.0%	0	0.0%
Marital Status	Married	0	0.0%	0	0.0%	0	0.0%	0	0.0%	40	72.7%
	Unmarried	240	100.0%	60	100.0%	40	100.0%	0	0.0%	0	0.0%
	Divorced	0	0.0%	0	0.0%	0	0.0%	5	100.0%	15	27.3%
Socioeconomic Class	Upper	0	0.0%	0	0.0%	20	50.0%	0	0.0%	12	21.8%
	Upper Middle	0	0.0%	0	0.0%	0	0.0%	5	100.0%	43	78.2%
	Lower Middle	60	25.0%	0	0.0%	0	0.0%	0	0.0%	0	0.0%
	Upper Lower	100	41.7%	0	0.0%	0	0.0%	0	0.0%	0	0.0%
	Lower	80	33.3%	60	100.0%	20	50.0%	0	0.0%	0	0.0%
Religion	Muslim	240	100.0%	60	100.0%	20	50.0%	0	0.0%	0	0.0%
	Christian	0	0.0%	0	0.0%	20	50.0%	0	0.0%	0	0.0%
	Hindu	0	0.0%	0	0.0%	0	0.0%	0	0.0%	48	87.3%
	Others	0	0.0%	0	0.0%	0	0.0%	5	100.0%	7	12.7%
Type of Family	Nuclear	240	100.0%	0	0.0%	0	0.0%	0	0.0%	0	0.0%
	Joint	0	0.0%	60	100.0%	40	100.0%	0	0.0%	0	0.0%
	Three Generation	0	0.0%	0	0.0%	0	0.0%	5	100.0%	55	100.0%

**Table 5:** Comparison of methods of contraception used in different demographies of participants The significant P-value is 0.05

Pearson Chi-Square Tests							
		Age of respondent	Education of respondent	Marital Status	Socioeconomic Class	Religion	Type of Family
Method of contraception used	Chi-square	6.311	318.265	472.727	587.879	732.955	800.000
	Difference of freedom	16	12	8	16	12	8
	P-value	.984	.000	.000	.000	.000	.000

The use of sanitary pads during menstrual bleeding is higher in educated women and married women with 68.8% of those who used pads being educated to the intermediate level and 62.5% being married (Table 6). The frequency of using cloth pieces was more prevalent in less educated (education up to eighth grade) and unmarried women, which is 50% and 87.5%, respectively. A similar prevalence is seen for cotton use, which is 64% and 50 % in women who studied up to eighth grade and women from the low socioeconomic class, respectively. Table 7 shows these results are found to be significant in chi-square analysis.

**Table 6:** Prevalence of different methods used during menstrual bleeding among participants

Population characteristics		What do you use during menstrual days?							
		Nothing		Cotton		Cloth pieces		Sanitary pads	
		Frequency	Percentage	Frequency	Percentage	Frequency	Percentage	Frequency	Percentage
Age of respondent	15-21	2	25.0%	49	24.5%	27	16.9%	5	15.6%
	22-28	3	37.5%	68	34.0%	51	31.9%	10	31.3%
	29-35	1	12.5%	33	16.5%	27	16.9%	6	18.8%
	36-42	1	12.5%	33	16.5%	28	17.5%	5	15.6%
	43-48	1	12.5%	17	8.5%	27	16.9%	6	18.8%
Education of respondent	up to 5th Grade	0	0.0%	26	13.0%	80	50.0%	10	31.3%
	Up to 8th Grade	0	0.0%	128	64.0%	80	50.0%	0	0.0%
	Matriculation	0	0.0%	0	0.0%	0	0.0%	0	0.0%
	Intermediate	8	100.0%	13	6.5%	0	0.0%	22	68.8%
	Bachelor	0	0.0%	33	16.5%	0	0.0%	0	0.0%
Marital Status	Married	0	0.0%	0	0.0%	20	12.5%	20	62.5%
	Unmarried	0	0.0%	200	100.0%	140	87.5%	0	0.0%
	Divorced	8	100.0%	0	0.0%	0	0.0%	12	37.5%
Socioeconomic Class	Upper	0	0.0%	0	0.0%	32	20.0%	0	0.0%
	Upper Middle	8	100.0%	0	0.0%	8	5.0%	32	100.0%
	Lower Middle	0	0.0%	60	30.0%	0	0.0%	0	0.0%
	Upper Lower	0	0.0%	100	50.0%	0	0.0%	0	0.0%
	Lower	0	0.0%	40	20.0%	120	75.0%	0	0.0%
Religion	Muslim	0	0.0%	200	100.0%	120	75.0%	0	0.0%
	Christian	0	0.0%	0	0.0%	20	12.5%	0	0.0%
	Hindu	0	0.0%	0	0.0%	20	12.5%	28	87.5%
	Others	8	100.0%	0	0.0%	0	0.0%	4	12.5%
Type of Family	Nuclear	0	0.0%	200	100.0%	40	25.0%	0	0.0%
	Joint	0	0.0%	0	0.0%	100	62.5%	0	0.0%
	Three Generation	8	100.0%	0	0.0%	20	12.5%	32	100.0%

**Table 7:** Comparison of menstrual bleeding management methods in different demographies of participants The significant p-value is 0.05

Pearson Chi-Square Tests							
		Age of respondent	Education of respondent	Marital Status	Socioeconomic Class	Religion	Type of Family
What do you use during menstrual days?	Chi-square	9.405	283.800	379.412	581.667	520.833	483.333
	Difference of freedom	12	9	6	12	9	6
	P-value	.668	.000	.000	.000	.000	.000

On asking about sexually transmitted diseases (STDs), in terms of their names and methods of transmission, married and upper middle class showed a prevalence of about 62.5% and 75%, respectively, and women with education up to intermediate level answered the same with 46.9%, while 75% of women belonging to the upper middle class were aware of it (Table 8). Unmarried women and women with low education (up to fifth and eighth grade) answered No with frequencies of 336 and 208, respectively. Table 9 shows these results are found to be significant on chi-square analysis.

**Table 8:** Prevalence of knowledge about sexually transmitted diseases in participants

Population characteristics		Do you know about sexually transmitted diseases?			
		Yes		No	
		Frequency	Percentage	Frequency	Percentage
Age of respondent	15-21	11	17.2%	72	21.4%
	22-28	21	32.8%	111	33.0%
	29-35	11	17.2%	56	16.7%
	36-42	10	15.6%	57	17.0%
	43-48	11	17.2%	40	11.9%
Education of respondent	up to 5th Grade	34	53.1%	82	24.4%
	Up to 8th Grade	0	0.0%	208	61.9%
	Matriculation	0	0.0%	0	0.0%
	Intermediate	30	46.9%	13	3.9%
	Bachelor	0	0.0%	33	9.8%
Marital status	Married	40	62.5%	0	0.0%
	Unmarried	4	6.3%	336	100.0%
	Divorced	20	31.3%	0	0.0%
Socioeconomic class	Upper	16	25.0%	16	4.8%
	Upper Middle	48	75.0%	0	0.0%
	Lower Middle	0	0.0%	60	17.9%
	Upper Lower	0	0.0%	100	29.8%
	Lower	0	0.0%	160	47.6%
Religion	Muslim	0	0.0%	320	95.2%
	Christian	4	6.3%	16	4.8%
	Hindu	48	75.0%	0	0.0%
	Others	12	18.8%	0	0.0%
Type of family	Nuclear	0	0.0%	240	71.4%
	Joint	4	6.3%	96	28.6%
	Three generation	60	93.8%	0	0.0%

**Table 9:** Comparison of knowledge about sexually transmitted diseases in different demographies of participants. The significant P-value is 0.05

Pearson Chi-Square Tests							
		Age of respondent	Education of respondent	Marital Status	Socioeconomic Class	Religion	Type of Family
Do you know about sexually transmitted diseases?	Chi-square	1.710	153.689	370.588	340.476	376.190	371.429
	Difference of freedom	4	3	2	4	3	2
	P-value	.789	.000	.000	.000	.000	.000

When asked about regular screening for STDs, 85.7% and 53.6% of women from the upper middle class and with education up to intermediate level answered Yes, respectively, and 46.55% of the lower class answered No (Table 10). These results are significant in chi-square analysis as shown in Table 11.

**Table 10:** Prevalence of having screening for sexually transmitted diseases in participants

Population characteristics		Do you have regular screening for sexually transmitted diseases?			
		Yes		No	
		Frequency	Percent	Frequency	Percent
Age of respondent	15-21	9	16.1%	74	21.5%
	22-28	19	33.9%	113	32.8%
	29-35	10	17.9%	57	16.6%
	36-42	9	16.1%	58	16.9%
	43-48	9	16.1%	42	12.2%
Education of respondent	up to 5th Grade	26	46.4%	90	26.2%
	Up to 8th Grade	0	0.0%	208	60.5%
	Matriculation	0	0.0%	0	0.0%
	Intermediate	30	53.6%	13	3.8%
	Bachelor	0	0.0%	33	9.6%
Marital Status	Married	36	64.3%	4	1.2%
	Unmarried	0	0.0%	340	98.8%
	Divorced	20	35.7%	0	0.0%
Socioeconomic Class	Upper	8	14.3%	24	7.0%
	Upper Middle	48	85.7%	0	0.0%
	Lower Middle	0	0.0%	60	17.4%
	Upper Lower	0	0.0%	100	29.1%
	Lower	0	0.0%	160	46.5%
Religion	Muslim	0	0.0%	320	93.0%
	Christian	0	0.0%	20	5.8%
	Hindu	44	78.6%	4	1.2%
	Others	12	21.4%	0	0.0%
Type of Family	Nuclear	0	0.0%	240	69.8%
	Joint	0	0.0%	100	29.1%
	Three Generation	56	100.0%	4	1.2%

**Table 11:** Comparison of having screening for sexually transmitted diseases in different demographies of participants The significant P-value is 0.05

Pearson Chi-Square Tests							
		Age of respondent	Education of respondent	Marital Status	Socioeconomic Class	Religion	Type of Family
Do you have regular screening for sexually transmitted diseases?	Chi-square	1.333	157.125	370.100	350.166	369.546	368.992
	Difference of freedom	4	3	2	4	3	2
	P-value	.856	.000	.000	.000	.000	.000

Table 12 shows that 37.5% of women with intermediate education accepted knowing about screening for cervical cancer. The prevalence is found to be about 50% in married women and 60% in the upper middle class. Of women with education up to eighth grade, 65% answered with No, and the prevalence is 50% for women from the lower socioeconomic class. Two hundred and forty women from nuclear-type families answered No. These results are found to be significant in chi-square analysis as seen in Table 13.

**Table 12:** Prevalence of knowledge about pap test for cervical cancer screening in participants.

		Do you know about pap test screening for cervical cancers?			
		Yes		No	
		Frequency	Percent	Frequency	Percent
Age of respondent	15-21	13	16.3%	70	21.9%
	22-28	27	33.8%	105	32.8%
	29-35	14	17.5%	53	16.6%
	36-42	13	16.3%	54	16.9%
	43-48	13	16.3%	38	11.9%
Education of respondent	up to 5th Grade	50	62.5%	66	20.6%
	Up to 8th Grade	0	0.0%	208	65.0%
	Matriculation	0	0.0%	0	0.0%
	Intermediate	30	37.5%	13	4.1%
	Bachelor	0	0.0%	33	10.3%
Marital Status	Married	40	50.0%	0	0.0%
	Unmarried	20	25.0%	320	100.0%
	Divorced	20	25.0%	0	0.0%
Socioeconomic Class	Upper	32	40.0%	0	0.0%
	Upper Middle	48	60.0%	0	0.0%
	Lower Middle	0	0.0%	60	18.8%
	Upper Lower	0	0.0%	100	31.3%
	Lower	0	0.0%	160	50.0%
Religion	Muslim	0	0.0%	320	100.0%
	Christian	20	25.0%	0	0.0%
	Hindu	48	60.0%	0	0.0%
	Others	12	15.0%	0	0.0%
Type of Family	Nuclear	0	0.0%	240	75.0%
	Joint	20	25.0%	80	25.0%
	Three Generation	60	75.0%	0	0.0%

**Table 13:** Comparison of knowledge about pap test screening in different demographies of participants. The significant P-value is 0.05

Pearson Chi-Square Tests							
		Age of respondent	Education of respondent	Marital Status	Socioeconomic Class	Religion	Type of Family
Do you know about pap test screening for cervical cancers?	Chi-square	2.002	165.512	282.353	400.000	400.000	300.000
	Difference of freedom	4	3	2	4	3	2
	P-value	.735	.000	.000	.000	.000	.000

When asked about how often the participants had pap test screening so far, 50% of women with up to eighth-grade education, 144 unmarried women (100%), and 41.7% of lower middle class answered never (Table 14). Ninety percent of married women, 60% of the upper middle class, and 90% of women with three-generation families mentioned having it regularly. These results are significant in chi-square analysis as seen in Table 15.

**Table 14:** Prevalence of having pap test for cervical cancer screening among participants.

		How often you had pap test screening so far?							
		Never		Do not know		Regularly		Irregularly	
		Frequency	Percent	Frequency	Percent	Frequency	Percent	Frequency	Percent
Age of respondent	15-21	39	27.1%	33	17.2%	7	17.5%	4	16.7%
	22-28	48	33.3%	63	32.8%	13	32.5%	8	33.3%
	29-35	24	16.7%	32	16.7%	7	17.5%	4	16.7%
	36-42	24	16.7%	33	17.2%	6	15.0%	4	16.7%
	43-48	9	6.3%	31	16.1%	7	17.5%	4	16.7%
Education of respondent	up to 5th Grade	26	18.1%	56	29.2%	34	85.0%	0	0.0%
	Up to 8th Grade	72	50.0%	136	70.8%	0	0.0%	0	0.0%
	Matriculation	0	0.0%	0	0.0%	0	0.0%	0	0.0%
	Intermediate	13	9.0%	0	0.0%	6	15.0%	24	100.0%
	Bachelor	33	22.9%	0	0.0%	0	0.0%	0	0.0%
Marital Status	Married	0	0.0%	0	0.0%	36	90.0%	4	16.7%
	Unmarried	144	100.0%	192	100.0%	4	10.0%	0	0.0%
	Divorced	0	0.0%	0	0.0%	0	0.0%	20	83.3%
Socioeconomic Class	Upper	0	0.0%	16	8.3%	16	40.0%	0	0.0%
	Upper Middle	0	0.0%	0	0.0%	24	60.0%	24	100.0%
	Lower Middle	60	41.7%	0	0.0%	0	0.0%	0	0.0%
	Upper Lower	84	58.3%	16	8.3%	0	0.0%	0	0.0%
	Lower	0	0.0%	160	83.3%	0	0.0%	0	0.0%
Religion	Muslim	144	100.0%	176	91.7%	0	0.0%	0	0.0%
	Christian	0	0.0%	16	8.3%	4	10.0%	0	0.0%
	Hindu	0	0.0%	0	0.0%	36	90.0%	12	50.0%
	Others	0	0.0%	0	0.0%	0	0.0%	12	50.0%
Type of Family	Nuclear	144	100.0%	96	50.0%	0	0.0%	0	0.0%
	Joint	0	0.0%	96	50.0%	4	10.0%	0	0.0%
	Three Generation	0	0.0%	0	0.0%	36	90.0%	24	100.0%

**Table 15:** Comparison of having pap test screening for cervical cancer in different demographies of participants. The significant P-value is 0.05

Pearson Chi-Square Tests							
		Age of respondent	Education of respondent	Marital Status	Socioeconomic Class	Religion	Type of Family
How often you had pap test screening so far?	Chi-square	11.981	360.864	659.765	718.000	536.333	489.600
	Difference of freedom	12	9	6	12	9	6
	P-value	.447	.000	.000	.000	.000	.000

Table 16 shows that 41.7% of married women, 33.3% of women in the upper class, and 50% of women in the upper middle class mentioned that they know about the human papillomavirus vaccine, while 68.4% of women with education up to eighth grade and 47.4% of lower class women did not know about it. These results are significant in chi-square analysis as seen in Table 17.

**Table 16:** Prevalence of knowledge about human papillomavirus vaccine in participants.

		Do you know about the Human papillomavirus vaccine?			
		yes		No	
		Frequency	Percent	Frequency	Percent
Age of respondent	15-21	16	16.7%	67	22.0%
	22-28	31	32.3%	101	33.2%
	29-35	17	17.7%	50	16.4%
	36-42	16	16.7%	51	16.8%
	43-48	16	16.7%	35	11.5%
Education of respondent	up to 5th Grade	66	68.8%	50	16.4%
	Up to 8th Grade	0	0.0%	208	68.4%
	Matriculation	0	0.0%	0	0.0%
	Intermediate	30	31.3%	13	4.3%
	Bachelor	0	0.0%	33	10.9%
Marital Status	Married	40	41.7%	0	0.0%
	Unmarried	36	37.5%	304	100.0%
	Divorced	20	20.8%	0	0.0%
Socioeconomic Class	Upper	32	33.3%	0	0.0%
	Upper Middle	48	50.0%	0	0.0%
	Lower Middle	0	0.0%	60	19.7%
	Upper Lower	0	0.0%	100	32.9%
	Lower	16	16.7%	144	47.4%
Religion	Muslim	16	16.7%	304	100.0%
	Christian	20	20.8%	0	0.0%
	Hindu	48	50.0%	0	0.0%
	Others	12	12.5%	0	0.0%
Type of Family	Nuclear	0	0.0%	240	78.9%
	Joint	36	37.5%	64	21.1%
	Three Generation	60	62.5%	0	0.0%

**Table 17:** Comparison of knowledge about human papillomavirus vaccine in different demographies of participants. The significant P-value is 0.05

Pearson Chi-Square Tests							
		Age of respondent	Education of respondent	Marital Status	Socioeconomic Class	Religion	Type of Family
Do you know about the Human papillomavirus vaccine?	Chi-square	2.624	194.309	223.529	321.053	316.667	273.684
	Difference of freedom	4	3	2	4	3	2
	P-value	.623	.000	.000	.000	.000	.000

Table 18 shows that 53.8% of women with education up to eighth grade did not have even a single dose of the human papillomavirus vaccine and 77.5% did not know. Twenty-four women with intermediate education had completed the course of that vaccine. Twenty-four women (40%) from the upper middle class had a complete course of this vaccine while 53.3% of upper-class women had a single dose of it, and 38.5% of the lower middle and 61.5% of the upper lower class did not have a single dose. These results are significant in chi-square analysis as seen in Table 19.

**Table 18:** Prevalence of having human papillomavirus vaccine doses among participants.

		How many Human papillomavirus vaccine doses you had so far?							
		Never		Do not know		1 dose		complete course	
		Frequency	Percent	Frequency	Percent	Frequency	Percent	Frequency	Percent
Age of respondent	15-21	42	26.9%	27	16.9%	10	16.7%	4	16.7%
	22-28	52	33.3%	53	33.1%	19	31.7%	8	33.3%
	29-35	26	16.7%	26	16.3%	11	18.3%	4	16.7%
	36-42	26	16.7%	27	16.9%	10	16.7%	4	16.7%
	43-48	10	6.4%	27	16.9%	10	16.7%	4	16.7%
Education of respondent	up to 5th Grade	26	16.7%	36	22.5%	54	90.0%	0	0.0%
	Up to 8th Grade	84	53.8%	124	77.5%	0	0.0%	0	0.0%
	Matriculation	0	0.0%	0	0.0%	0	0.0%	0	0.0%
	Intermediate	13	8.3%	0	0.0%	6	10.0%	24	100.0%
	Bachelor	33	21.2%	0	0.0%	0	0.0%	0	0.0%
Marital Status	Married	0	0.0%	0	0.0%	36	60.0%	4	16.7%
	Unmarried	156	100.0%	160	100.0%	24	40.0%	0	0.0%
	Divorced	0	0.0%	0	0.0%	0	0.0%	20	83.3%
Socioeconomic Class	Upper	0	0.0%	0	0.0%	32	53.3%	0	0.0%
	Upper Middle	0	0.0%	0	0.0%	24	40.0%	24	100.0%
	Lower Middle	60	38.5%	0	0.0%	0	0.0%	0	0.0%
	Upper Lower	96	61.5%	4	2.5%	0	0.0%	0	0.0%
	Lower	0	0.0%	156	97.5%	4	6.7%	0	0.0%
Religion	Muslim	156	100.0%	160	100.0%	4	6.7%	0	0.0%
	Christian	0	0.0%	0	0.0%	20	33.3%	0	0.0%
	Hindu	0	0.0%	0	0.0%	36	60.0%	12	50.0%
	Others	0	0.0%	0	0.0%	0	0.0%	12	50.0%
Type of Family	Nuclear	156	100.0%	84	52.5%	0	0.0%	0	0.0%
	Joint	0	0.0%	76	47.5%	24	40.0%	0	0.0%
	Three Generation	0	0.0%	0	0.0%	36	60.0%	24	100.0%

**Table 19:** Comparison of having human papillomavirus vaccine in different demographies of participants. The significant P-value is 0.05

Pearson Chi-Square Tests							
		Age of respondent	Education of respondent	Marital Status	Socioeconomic Class	Religion	Type of Family
How many Human Papillomavirus vaccine doses you had so far?	Chi-square	12.917	405.780	539.059	864.804	558.667	420.300
	Difference of freedom	12	9	6	12	9	6
	P-value	.375	.000	.000	.000	.000	.000

Table 20 shows that 92.5% of women with education up to eighth grade had one to two deliveries at home and 68.8% of women with education up to fifth grade had three to four deliveries at home. Fifty percent of both lower and upper lower class had one to two deliveries at home. Twelve women from the upper middle class had all of their deliveries at home, and 20 had five-six deliveries at home. These results are significant in chi-square analysis as seen in Table 21.

**Table 20:** Prevalence of having home deliveries in participants.

		How many home deliveries you have so far?									
		Do not know		1-2		3-4		5-6		All of them	
		Frequency	Percent	Frequency	Percent	Frequency	Percent	Frequency	Percent	Frequency	Percent
Age of respondent	15-21	24	30.0%	32	20.0%	21	16.4%	4	20.0%	2	16.7%
	22-28	27	33.8%	53	33.1%	41	32.0%	7	35.0%	4	33.3%
	29-35	13	16.3%	27	16.9%	22	17.2%	3	15.0%	2	16.7%
	36-42	13	16.3%	27	16.9%	22	17.2%	3	15.0%	2	16.7%
	43-48	3	3.8%	21	13.1%	22	17.2%	3	15.0%	2	16.7%
Education of respondent	up to 5th Grade	18	22.5%	8	5.0%	88	68.8%	2	10.0%	0	0.0%
	Up to 8th Grade	20	25.0%	148	92.5%	40	31.3%	0	0.0%	0	0.0%
	Matriculation	0	0.0%	0	0.0%	0	0.0%	0	0.0%	0	0.0%
	Intermediate	9	11.3%	4	2.5%	0	0.0%	18	90.0%	12	100.0%
	Bachelor	33	41.3%	0	0.0%	0	0.0%	0	0.0%	0	0.0%
Marital Status	Married	0	0.0%	0	0.0%	28	21.9%	12	60.0%	0	0.0%
	Unmarried	80	100.0%	160	100.0%	100	78.1%	0	0.0%	0	0.0%
	Divorced	0	0.0%	0	0.0%	0	0.0%	8	40.0%	12	100.0%
Socioeconomic Class	Upper	0	0.0%	0	0.0%	32	25.0%	0	0.0%	0	0.0%
	Upper Middle	0	0.0%	0	0.0%	16	12.5%	20	100.0%	12	100.0%
	Lower Middle	60	75.0%	0	0.0%	0	0.0%	0	0.0%	0	0.0%
	Upper Lower	20	25.0%	80	50.0%	0	0.0%	0	0.0%	0	0.0%
	Lower	0	0.0%	80	50.0%	80	62.5%	0	0.0%	0	0.0%
Religion	Muslim	80	100.0%	160	100.0%	80	62.5%	0	0.0%	0	0.0%
	Christian	0	0.0%	0	0.0%	20	15.6%	0	0.0%	0	0.0%
	Hindu	0	0.0%	0	0.0%	28	21.9%	20	100.0%	0	0.0%
	Others	0	0.0%	0	0.0%	0	0.0%	0	0.0%	12	100.0%
Type of Family	Nuclear	80	100.0%	160	100.0%	0	0.0%	0	0.0%	0	0.0%
	Joint	0	0.0%	0	0.0%	100	78.1%	0	0.0%	0	0.0%
	Three Generation	0	0.0%	0	0.0%	28	21.9%	20	100.0%	12	100.0%

**Table 21:** Comparison of having home deliveries in different demographies of participants. The significant P-value is 0.05

Pearson Chi-Square Tests							
		Age of respondent	Education of respondent	Marital Status	Socioeconomic Class	Religion	Type of Family
How many home deliveries you have so far?	Chi-square	12.113	559.253	411.515	688.333	642.708	566.667
	Difference of freedom	16	12	8	16	12	8
	P-value	.736	.000	.000	.000	.000	.000

Table 22 shows that 64.3% of women with education up to fifth grade and 28.6% of women with education up to eighth grade had all their deliveries at a hospital. This was also true of 22.9% of women from the upper class and 20% of the upper middle class. Thirty-three women (41.3%) with bachelor’s education had one to two deliveries in the hospital. These results are found to be significant in chi-square analysis as seen in Table 23.

**Table 22:** Prevalence of having hospital or maternity home deliveries in participants.

		How many hospital or maternity home deliveries you have so far?									
		Do not know		1-2		3-4		5-6		All of them	
		Frequency	Percent	Frequency	Percent	Frequency	Percent	Frequency	Percent	Frequency	Percent
Age of respondent	15-21	22	18.3%	24	30.0%	10	25.0%	4	20.0%	23	16.4%
	22-28	40	33.3%	27	33.8%	13	32.5%	7	35.0%	45	32.1%
	29-35	20	16.7%	13	16.3%	7	17.5%	3	15.0%	24	17.1%
	36-42	20	16.7%	13	16.3%	7	17.5%	3	15.0%	24	17.1%
	43-48	18	15.0%	3	3.8%	3	7.5%	3	15.0%	24	17.1%
Education of respondent	up to 5th Grade	0	0.0%	18	22.5%	8	20.0%	0	0.0%	90	64.3%
	Up to 8th Grade	120	100.0%	20	25.0%	28	70.0%	0	0.0%	40	28.6%
	Matriculation	0	0.0%	0	0.0%	0	0.0%	0	0.0%	0	0.0%
	Intermediate	0	0.0%	9	11.3%	4	10.0%	20	100.0%	10	7.1%
	Bachelor	0	0.0%	33	41.3%	0	0.0%	0	0.0%	0	0.0%
Marital Status	Married	0	0.0%	0	0.0%	0	0.0%	0	0.0%	40	28.6%
	Unmarried	120	100.0%	80	100.0%	40	100.0%	0	0.0%	100	71.4%
	Divorced	0	0.0%	0	0.0%	0	0.0%	20	100.0%	0	0.0%
Socioeconomic Class	Upper	0	0.0%	0	0.0%	0	0.0%	0	0.0%	32	22.9%
	Upper Middle	0	0.0%	0	0.0%	0	0.0%	20	100.0%	28	20.0%
	Lower Middle	0	0.0%	60	75.0%	0	0.0%	0	0.0%	0	0.0%
	Upper Lower	40	33.3%	20	25.0%	40	100.0%	0	0.0%	0	0.0%
	Lower	80	66.7%	0	0.0%	0	0.0%	0	0.0%	80	57.1%
Religion	Muslim	120	100.0%	80	100.0%	40	100.0%	0	0.0%	80	57.1%
	Christian	0	0.0%	0	0.0%	0	0.0%	0	0.0%	20	14.3%
	Hindu	0	0.0%	0	0.0%	0	0.0%	8	40.0%	40	28.6%
	Others	0	0.0%	0	0.0%	0	0.0%	12	60.0%	0	0.0%
Type of Family	Nuclear	120	100.0%	80	100.0%	40	100.0%	0	0.0%	0	0.0%
	Joint	0	0.0%	0	0.0%	0	0.0%	0	0.0%	100	71.4%
	Three Generation	0	0.0%	0	0.0%	0	0.0%	20	100.0%	40	28.6%

**Table 23:** Comparison of having hospital deliveries in different demographies of participants. The significant P-value is 0.05

Pearson Chi-Square Tests							
		Age of respondent	Education of respondent	Marital Status	Socioeconomic Class	Religion	Type of Family
How many hospital or maternity home deliveries you have so far?	Chi-square	14.099	489.876	480.672	685.714	376.190	495.238
	Difference of freedom	16	12	8	16	12	8
	P-value	.591	.000	.000	.000	.000	.000

On asking about follow-up visits after each delivery, 112 women with education up to eighth grade admitted to having it regularly and 96 women irregularly (Table 24). Sixty women with education up to fifth grade never had a follow-up while 35.7% from lower middle and 59.5% from the upper lower class had it regularly. The prevalence of having regular follow-up visits is higher in nuclear families compared to joint and three-generation families. These results are significant in chi square analysis as seen in Table 25.

**Table 24:** Prevalence of follow-ups after each delivery among participants.

		How often follow-ups you had after each delivery?							
		Do not know		Regularly		Irregularly		Never	
		Frequency	Percent	Frequency	Percent	Frequency	Percent	Frequency	Percent
Age of respondent	15-21	9	17.3%	20	16.7%	44	26.2%	10	16.7%
	22-28	17	32.7%	38	31.7%	57	33.9%	20	33.3%
	29-35	9	17.3%	21	17.5%	27	16.1%	10	16.7%
	36-42	8	15.4%	21	17.5%	28	16.7%	10	16.7%
	43-48	9	17.3%	20	16.7%	12	7.1%	10	16.7%
Education of respondent	up to 5th Grade	22	42.3%	8	6.7%	26	15.5%	60	100.0%
	Up to 8th Grade	0	0.0%	112	93.3%	96	57.1%	0	0.0%
	Matriculation	0	0.0%	0	0.0%	0	0.0%	0	0.0%
	Intermediate	30	57.7%	0	0.0%	13	7.7%	0	0.0%
	Bachelor	0	0.0%	0	0.0%	33	19.6%	0	0.0%
Marital Status	Married	32	61.5%	0	0.0%	0	0.0%	8	13.3%
	Unmarried	0	0.0%	120	100.0%	168	100.0%	52	86.7%
	Divorced	20	38.5%	0	0.0%	0	0.0%	0	0.0%
Socioeconomic Class	Upper	4	7.7%	0	0.0%	0	0.0%	28	46.7%
	Upper Middle	48	92.3%	0	0.0%	0	0.0%	0	0.0%
	Lower Middle	0	0.0%	0	0.0%	60	35.7%	0	0.0%
	Upper Lower	0	0.0%	0	0.0%	100	59.5%	0	0.0%
	Lower	0	0.0%	120	100.0%	8	4.8%	32	53.3%
Religion	Muslim	0	0.0%	120	100.0%	168	100.0%	32	53.3%
	Christian	0	0.0%	0	0.0%	0	0.0%	20	33.3%
	Hindu	40	76.9%	0	0.0%	0	0.0%	8	13.3%
	Others	12	23.1%	0	0.0%	0	0.0%	0	0.0%
Type of Family	Nuclear	0	0.0%	72	60.0%	168	100.0%	0	0.0%
	Joint	0	0.0%	48	40.0%	0	0.0%	52	86.7%
	Three Generation	52	100.0%	0	0.0%	0	0.0%	8	13.3%

**Table 25:** Comparison of having follow-ups after each delivery in different demographies of participants. The significant P-value is 0.05

Pearson Chi-Square Tests							
		Age of respondent	Education of respondent	Marital Status	Socioeconomic Class	Religion	Type of Family
How often followups you had after each delivery?	Chi-square	11.603	410.157	353.279	860.982	472.274	562.844
	Difference of freedom	12	9	6	12	9	6
	Sig.	.478	.000	.000	.000	.000	.000

## Discussion

The prevalence of knowledge about the vaccination of HPV was found to be less in women from low socioeconomic status (16%), as compared to women from upper socioeconomic status, which was 33.3%. This can be attributed to poor knowledge, which translates into poor practices. A review article highlighted the finding that bad odor contributed to fear, embarrassment, and distress among school-going girls; it hampered freedom to participate in daily activities during menstruation and is an insignia of proper menstrual practice [14]. Fortunately, as per our study, the condition in Pakistan is still better than in other Asian countries. For instance, in Nepal, very strict ritual seclusion of “Chaupaudi” is still practiced where women are ostracized during the entire period of menstruation [14].

Limited availability of soap and water deters some women from low socioeconomic backgrounds from regular washing of the groin during menses [15]. It has been suggested that women with dysmenorrhea or other menstrual disorders were often hesitant to discuss matters pertaining to sexual health owing to their warped cultural values and many found the healthcare providers to be unsupportive [15]

A systematic review was conducted to assess the effectiveness of "hardware intervention", that is, the provision of absorbing materials to address the material deprivations and access to water, sanitation, and hygiene (WASH) facilities [16]. A moderate non-significant effect was observed when reusable homemade and disposable sanitary pads were provided. Nevertheless, it is still believed that the institutional availability of pads can benefit young girls hailing from a low socioeconomic background, as a study in Ghana revealed that school attendance rose by 9% after five months of provision of disposable sanitary pads [16]. The same is the case in our study in which only 31.3% of women from low education backgrounds use sanitary pads and the prevalence is even less in lower socioeconomic classes. 

Menses leave policy has been implemented in the United Kindom, India, and Australia where menstruators are exempted from working while they are experiencing severe pain or discomfort [14]. This can empower women as it is an acknowledgment of their physiological process. Such policies can also be introduced in Pakistan to facilitate the healthcare workers and the general working force.

In India, the majority of women are excluded from religious gatherings and, in rural areas, women are restricted from even entering the kitchen during menstrual bleeding days [17]. The superstition of the association of menstruation with evil spirits is particularly prevalent in Asia. A menstruating woman deemed impure is more vulnerable to getting possessed by demons and, hence, some women bury the clothes used during menstruation [17].

Akbarzadeh et al. reported a significant association between age at menarche and dysmenorrhea onset [18]. On the other hand, Kural et al. could not find such a correlation [19]. The different results may be attributed to the differences between nutritional habits, public health, geographic location, and cultural factors in the studies. According to the literature, dysmenorrhea usually begins within one to two years after menarche [20]. This indicates the importance of educating adolescent girls at the age of menarche about dysmenorrhea. In our study, dysmenorrhea prevalence is found to be 60.0% for at least two to three days in a regular menstrual cycle in participants. This finding is consistent with the results previously reported in the literature.

In a study by Chen and Chen from the United States, adolescents were observed to largely have moderate-to-severe menstrual cramps [20]. In a study by Gun et al., dysmenorrhea began with menstruation in 39.9%, one to two hours before menstruation in 37.2%, and a few days prior to menstruation in 22.9% of the participants [21]. In our study, 60% had menarche at the age of 12 years with 77% of participants having a duration of the menstrual cycle of 25 to 28 days, 70.5% having four to five days of menstrual bleeding, and 14% having at least one episode of irregular bleeding in a menstrual cycle.

Most developing counties, however, have been unable to implement comprehensive Pap smear screening-based programs. In countries where Pap smear screening is available, it often is accessible to only a small proportion of women through private healthcare providers, or it is offered primarily to young women through maternal or child health clinics or family planning clinics where the population being screened generally is not at high risk [22]. These approaches have had little effect on morbidity and mortality and generally are not as cost-effective as centrally organized screening programs implemented by the public sector [23]. STIs have a great impact on the health of populations worldwide. These may be contracted by people of any age, race, or social standing, and their early diagnosis and treatment are necessary to avoid propagation. Sexual education is fundamental to STI prevention [24].

The high number of participants with a lack of knowledge about healthy reproductive practices in our study can be explained by the fact that the majority of Pakistani women consult family elders, local hakeem (homeopathic doctors), or daies (untrained local women who assist in the birth of children) for their decision about reproductive health. People also sometimes turn to pharmacies or traditional healers instead of healthcare facilities, and self-medication or alternative therapies can make STDs worse or better. Many people seek medical advice when the pain is unbearable. Therefore, the differences in STI prevalence between upper and lower classes, as well as under and well-educated classes can be attributed to various factors like family norms, social beliefs, and level of awareness of complications between the study populations.

The factors that determine health behaviors in Pakistan can be seen in various physical, socio-economic, cultural, and political contexts. Religious and social ethics discourage open discussion of sexual matters. Women's low social status limits their economic opportunities, and women can trade sex for money or other forms of support. Poor health services provide little for the prevention and treatment of STDs. Various factors, including proximity, affordability, availability, family pressure, and strong community opinion, lead to self-care and consultation with traditional healers, hakeems, or even quacks [25].

Long-held misconceptions continue to contribute to the nationwide neglect of treatment and prevention of STIs. Immediate STD detection, prevention, and STD-related counseling in clinics for vulnerable groups, as well as raising awareness, should be the basic pillars of the health policy of the public and private health sectors in Pakistan.

Limitations of the study

It is important to note that this should not be considered an accurate predictor of knowledge, attitude, and practice related to menstrual hygiene in the female population of the whole country. Second, our study had a narrow coverage of the socioeconomic classes, with most participants belonging to the lower and middle classes. Third, using a specific type of contraceptive also depends on other factors like medical indication or contraindication, cultural, and religious beliefs, but in this study, the research mainly shows the impact of education and socioeconomic levels on knowledge about different types of contraceptives and their usage. The course should be more varied and conducted with a larger sample size in the future to more comprehensively assess women's practices in a geographic area. The questionnaire was self-designed and many commonly believed myths may have been overlooked, and recall bias may have occurred answering some questions in the survey.

## Conclusions

Knowledge and practice about reproductive health as well as awareness about the HPV vaccine and Pap smear test were found to be low in the participants with low education levels. Moreover, in these rural areas, the reported use of contraceptives was low among women with education up to the eighth grade and reliable methods of contraception were more prevalent in women with an education of intermediate level or above. Women from the upper and upper middle class have more awareness and knowledge about STDs and their screening methods and cervical cancer screening and these results were found to be significant. The trend of having home deliveries is found to be more in women with less education. and hospital deliveries are more prevalent in women with education up to an intermediate level or above.

To overcome all of these, early education regarding menstrual hygiene and regular screening of cervical cancer and STDs should be provided to women of reproductive age in these rural areas using various learning materials. It's also very important to get teenage women involved in health education, both in high school and college, to improve the situation.
